# Evolutionary Theories and Men's Preferences for Women's Waist-to-Hip Ratio: Which Hypotheses Remain? A Systematic Review

**DOI:** 10.3389/fpsyg.2019.01221

**Published:** 2019-06-04

**Authors:** Jeanne Bovet

**Affiliations:** Stony Brook University, Stony Brook, NY, United States

**Keywords:** mate choice, attractiveness, evolutionary hypotheses, WHR, mate value, reproductive success, fertility

## Abstract

Over the last 25 years, a large amount of research has been dedicated to identifying men's preferences for women's physical features, and the evolutionary benefits associated with such preferences. Today, this area of research generates substantial controversy and criticism. I argue that part of the crisis is due to inaccuracies in the evolutionary hypotheses used in the field. For this review, I focus on the extensive literature regarding men's adaptive preferences for women's waist-to-hip ratio (WHR), which has become a classic example of the just-so storytelling contributing to the general mistrust toward evolutionary explanations of human behavior. The issues in this literature originate in the vagueness and incompleteness of the theorizing of the evolutionary mechanisms leading to mate preferences. Authors seem to have rushed into testing and debating the effects of WHR on women's attractiveness under various conditions and using different stimuli, without first establishing (a) clear definitions of the central evolution concepts (e.g., female mate value is often reduced to an imprecise concept of “health-and-fertility”), and (b) a complete overview of the distinct evolutionary paths potentially at work (e.g., focusing on fecundability while omitting descendants' quality). Unsound theoretical foundations will lead to imprecise predictions which cannot properly be tested, thus ultimately resulting in the premature rejection of an evolutionary explanation to human mate preferences. This paper provides the first comprehensive review of the existing hypotheses on why men's preferences for a certain WHR in women might be adaptive, as well as an analysis of the theoretical credibility of these hypotheses. By dissecting the evolutionary reasoning behind each hypothesis, I show which hypotheses are plausible and which are unfit to account for men's preferences for female WHR. Moreover, the most cited hypotheses (e.g., WHR as a cue of health or fecundity) are found to not necessarily be the ones with the strongest theoretical support, and some promising hypotheses (e.g., WHR as a cue of parity or current pregnancy) have seemingly been mostly overlooked. Finally, I suggest some directions for future studies on human mate choice, to move this evolutionary psychology literature toward a stronger theoretical foundation.

## Introduction

The ratio between the waist and the hips circumferences (Waist-to-Hip Ratio, or WHR) is a physical characteristic often used as an example to show that evolution shaped human mate preferences. It is also an example of just-so storytelling in evolutionary psychology. In 1993, Devendra Singh suggested that WHR represents a strong predictor of women's physical attractiveness (Singh, 1993a). He also argued that men's preference for a mate with a low WHR is adaptive, because a low WHR reflects a woman's high mate value. But what exactly is this “mate value”? During the past 25 years, the evolutionary literature on WHR and women's attractiveness has flourished, but the definition of this “mate value” is rarely expressed. In evolutionary biology, mate value is attached to the concept of reproductive success: a woman with a high mate value will increase the reproductive success of her mate(s). An increase in reproductive success is characterized by an increased number of descendants in next generations and can be achieved in various ways. First, survival until reproduction is indispensable. Second, the number of children born during an individual's lifespan is also crucial. But the survival and the quality of these children will directly impact their own reproductive success, and hence the number of grandchildren in the next generation, thus ultimately influencing the reproductive success of the grandparents. In short, a woman has higher value as a potential mate if she increases the number and quality of descendants a man will have (including the ones he has with other women). The question then is which of these components of reproductive success are actually linked to a mate's WHR? To answer this, I assemble the numerous hypotheses exposed since the idea of the WHR as an indicator of women's mate value was first suggested in 1993. These hypotheses are examined to determine which of the characteristics linked to WHR are most likely, in theory, to be translated into an increase in the reproductive success of the woman's mate.

The objective of this review is 2-fold. The first goal is to gather and pool all the existing evolutionary hypotheses regarding men's preferences for a certain (low, high or average) WHR. There are many reviews about men's preferences for women's WHR, but this is the first exhaustive review of the hypotheses mentioned in these studies. The second purpose of this paper is an in-depth theoretical examination of these hypotheses, which are often only briefly justified and, in some cases, have never been properly developed.

Most of the debate around WHR and attractiveness has centered on two other questions: “Is the preference for a low WHR universal?” and “Is WHR the best predictor of the attractiveness of women's bodies?” I will not address these two questions extensively here (it is beyond the scope of this paper), but a brief commentary seems necessary at this point. A preference for a relatively low WHR (i.e., low relatively to men's WHR, or low relatively to the average female WHR) has been observed in a large number of studies, including a wide range of populations and methods. With that in mind, results show that there is some variation in what is the exact value of the ideal WHR [reviewed in Brooks et al. (2015) and Cashdan (2008)]. The second debate concerns WHR as the “best” predictor for attractiveness. Authors have debated whether WHR or BMI is the best predictor of attractiveness and mate value (Tassinary and Hansen, 1998; Tovée et al., 1999; Furnham et al., 2005; Cornelissen et al., 2009a,b). As could be expected, the results vary according to the population and stimuli used. Other measurements have also been proposed to replace WHR (for example, hip or waist size alone, abdominal depth or waist/stature ratio: Brooks et al., 2010, 2015; Lassek and Gaulin, 2016). The objective of this paper is not to decide if WHR is the best measure of physical attractiveness or if the ideal WHR is universal or not. For our purposes, it is sufficient to note that the effect of WHR on attractiveness is widespread (even if the value of the preferred WHR varies), and large enough to warrant questions about its possible adaptive basis.

## Materials and Methods

**Figure 1:**
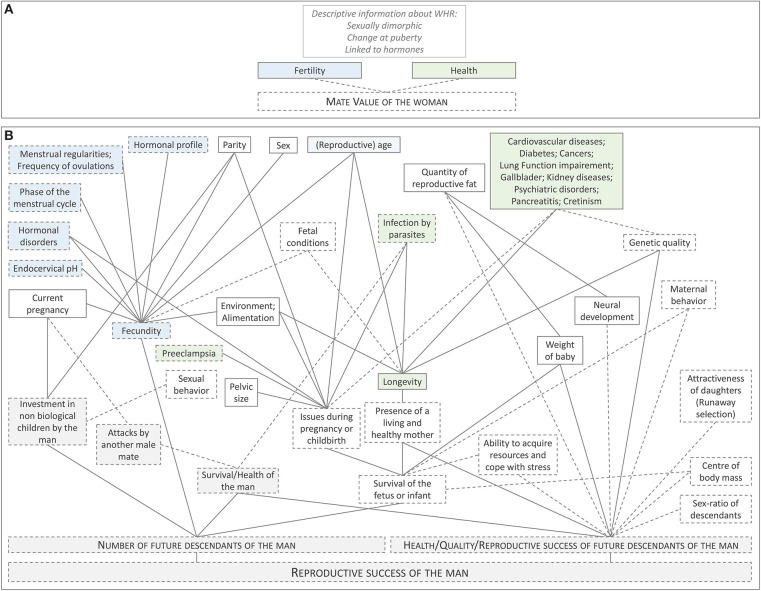
Two theoretical frameworks explaining men's adaptive preferences for women's WHR. **(A)** Example of a vague theoretical explanation often found in the literature. WHR is assumed to be a cue of women's health and fertility, which supposedly translate into women's “mate value.” Some descriptive information of WHR is sometimes added to the theory (e.g., WHR is sexually dimorphic), but without any explicit link to mate value. **(B)** A more complex but more accurate theoretical explanation, including all the different hypotheses found in the literature. Each box represents a characteristic linked to women's WHR. The diagram illustrates their potential links to the reproductive success of the man (the woman's mate). The boxes with a gray background directly concern the man. All the other boxes relate to the woman's characteristics. Characteristics related to women's fertility (as usually defined in this literature) are represented in *blue*. In *green*: characteristics related to women's health. The lines connecting the boxes represent correlations, without implying any causality. Dotted lines indicate that empirical evidences for the correlation are scarce. Dotted frames indicate that evidences linking the characteristic to women's WHR are scarce. The links between each characteristic are not represented on the diagram. For example, parity is obviously correlated with a woman's age, but this correlation is not illustrated here (each characteristic is supposed to be correlated to WHR, controlling for other characteristics).

The dataset encompasses any articles and book chapters addressing men's preferences for women's WHR, based on an evolutionary approach (see the Supplementary Material for the details). The final dataset consists of 104 papers from 58 different first authors, including 13 review papers and chapters from 1993 to 2017. All the hypotheses concerning men's adaptive preferences toward women's WHRs, waist size or hip size are collected (see Figure 1 and Supplementary Table S1).

See the Supplementary Material for the details about the methodology used in the collection and the selection of hypotheses.

## Results

In the following sections, I review each hypothesis found in the literature to see if it could, in theory, support an adaptive role of the preference for a low WHR. For a hypothesis to be plausible, two steps are required: 1) Correlation with WHR: first, WHR needs to be correlated with the biological trait of interest (Is WHR associated with the nominated characteristic in the population?). This correlation needs to be strong enough, such that a detectable variation of WHR attractiveness translates into a significant variation of the hypothesized trait; 2) Effect on the man's reproductive success: second, the nominated characteristic should be associated with a potential increase of reproductive success (meaning more descendants, and higher quality descendants) for the individual who chooses a mate carrying this characteristic. A dispensable third step can be added: 3) Perception of the characteristic using WHR: do people use the WHR to assess the nominated characteristic (Are people conscious of the link between WHR and the characteristic)? Importantly, this third step is not mandatory for the hypothesis to be valid, as people do not need to be conscious of the biological consequences of their preferences for them to have an effect. In other words, a preference for a trait can perfectly evolve when individuals do not suspect that this trait is a cue of something else. For example, a preference for sweet taste evolved in our ancestors without them knowing that it was a cue of a source of readily available energy. However, this third step can represent additional support in favor of the hypothesis and help us understand the mechanisms behind mate preferences.

### Cue of Biological Sex

**Figure 2:**
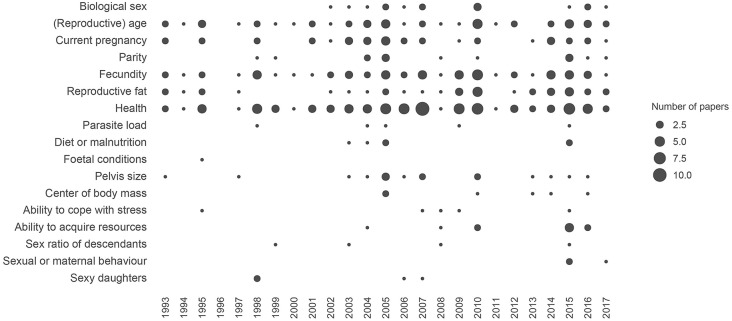
Temporal pattern of references to the different hypotheses of men's adaptive preferences for women's WHR in the literature. This figure includes scientific papers and chapters exploring the relationships between WHR and female attractiveness from an evolutionary perspective. The size of the circles represents the number of papers citing each hypothesis in a given year. See the Supplementary Material for the details of this figure.

According to this hypothesis, WHR would be a way to detect the biological sex of a potential mate. The first mention of this straightforward hypothesis appears relatively late, almost 10 years after the first paper about adaptive preferences for WHR (Tovée et al., 2002, see Figure 2), and is present in only 14% of the papers (see Supplementary Material).

#### Correlation With WHR

WHR is sexually dimorphic in the human species. A significant difference between men's and women's WHR has been found in all the populations where it has been investigated (Leibel et al., 1989; Björntorp, 1991, 1993; Marti et al., 1991; Beall and Goldstein, 1992; Ley et al., 1992). The size of the difference between sexes varies between populations, but no culture has been found where men have a lower average WHR than women. Thus, WHR is a reliable cue of biological sex.

However, there are many other traits which are sexually dimorphic in humans and which can be used to assess biological sex: height, shoulder-to-hip ratio, hair, facial traits, breast, genital, voice, and so on (Wenzlaff et al., 2018). Consequently, people can identify biological sex without using WHR. And because WHR is not indispensable to asses sex, the selective advantage of a preference for a low WHR as a way to assess biological sex is reduced (Iwasa and Pomiankowski, 1994; Bro-Jørgensen, 2010). On the other hand, the “redundant signaling” hypothesis (or “back-up signal” hypothesis) claims that multiple cues conveying similar information (the biological sex in the present case) compensate for errors during information coding (Moller and Pomiankowski, 1993; Johnstone, 1996). In other words, multiple cues serve as a back-up signal that ensures a low rate of mate choice errors (Bro-Jørgensen, 2010; Abend et al., 2015). Moreover, when the ability to detect different cues varies with environmental conditions or distances, individuals may pay attention to different cues under different conditions (Candolin, 2003). More experiments are required to measure by how much and when WHR does improve the detection of biological sex, in addition to other sexually dimorphic features.

#### Effect on the Man's Reproductive Success

Obviously, it is necessary to copulate with the opposite sex to increase the number of descendants. The problem in this case is not the effect of the characteristic (biological sex) on the individual's reproductive success, but the non-uniqueness of the cue. Thus, unless we discover evidence supporting the “redundant signaling” hypothesis, the sexual dimorphism of WHR may have contributed to the selection of men's preference for a low WHR but was probably not the only selective force involved.

#### Perception of the Characteristic Using WHR

People use WHR to assess individuals' biological sex, and a lower WHR is strongly associated with figures being perceived as female (Johnson and Tassinary, 2005; Johnson et al., 2010; Saunders et al., 2010; Pazhoohi and Liddle, 2012). People are able to detect sex using WHR when other cues of sex are unavailable (Pazhoohi and Liddle, 2012) but, as stated earlier, we need to measure the accuracy of this detection with and without the use of WHR. This would give us an indication of the strength of the selection on the use of WHR as a cue of biological sex.

### Cue of (Reproductive) Age

This hypothesis, already referred to in the first paper on the topic (Singh, 1993a, see Figure 2), is found in 43% of the papers. According to this hypothesis, WHR would be an indicator of chronological or reproductive age.

#### Correlation With WHR

WHR is high in early childhood (and similar between boys and girls) and drops around the onset of puberty for women. Then, women's WHR increases from the peak of fertility (in the 20's). This general age pattern is observed in many countries, including non-western and non-industrial populations (Rimm et al., 1988; Leibel et al., 1989; Seidell et al., 1990; Beall and Goldstein, 1992; Ley et al., 1992; Björntorp, 1993; Casey et al., 1994; Sugiyama, 2004; Bohler et al., 2010; Brooks et al., 2010; Bacopoulou et al., 2015; Butovskaya et al., 2017). Finally, WHR might also be a cue of menopause (end of reproductive age, independently of chronological age; Kirschner and Samojlik, 1991; Bjorkelund et al., 1996; Tchernof and Poehlman, 1998), but several other studies find no effect of menopausal status on WHR (Lanska et al., 1985; Tonkelaar et al., 1989; Seidell et al., 1990; Troisi et al., 1995; Tchernof and Poehlman, 1998; Sugiyama, 2004).

To sum up, WHR is a reliable cue of the start of women's reproductive capacity (menarche and puberty). WHR is also a reliable indicator of women's age after puberty, and maybe of menopause. However, as with biological sex (although to a lesser extent), individuals can rely on other cues to assess age (the face, for example, and menarche is also linked to an increase in breast size). The redundancy of the cue decreases the selective advantage of the preference for a low WHR as cue of age (Iwasa and Pomiankowski, 1994; Bro-Jørgensen, 2010). Nevertheless, according to the “redundant signaling” hypothesis, using several cues simultaneously, including WHR, may increase the precision of the age estimation (Johnstone, 1996; Bro-Jørgensen, 2010; Abend et al., 2015). Alternatively, the use of redundant cues may reduce time and energy spent inspecting mates, make mate assessment possible under different conditions (Rowe, 1999; Candolin, 2003), or make it more difficult for women to cheat about their actual age (Candolin, 2003).

#### Effect on the Man's Reproductive Success

Fecundability (the ability to become pregnant) is age-dependent for women. Fecundability is null before menarche, increases from puberty, peaks in mid-twenties on average, and then decreases until menopause, the end of the reproductive window (Menken and Larsen, 1986; Weinstein et al., 1990; Dunson et al., 2002; Wallace and Kelsey, 2010). In addition, the risks of complications during pregnancy and childbirth are also related to age (more issues when very young, and after 30, even when controlling for parity; Naeye, 1983; Fretts et al., 1995; Amarin and Akasheh, 2001). Thus, the choice of a mate around the peak of fertility (for short-term relationships), or before (for long-term relationships), will increase the number of potential descendants a man can sire with this mate, and thus his reproductive success.

However, because WHR is not the only cue of women's age, the correlation between age and WHR may have contributed to the selection and/or maintenance of men's preferences for a low WHR, but it is unlikely to be sufficient on its own, unless the use of WHR in addition to other redundant cues increases the precision of the age estimation in a way which would confer a selective advantage to the men making this estimation. More investigation is necessary to explore the role of the back-up signal hypothesis in this case.

#### Perception of the Characteristic Using WHR

People seem to use WHR to assess women's age, and a low WHR tends to be associated with perceptions of youthfulness (Singh, 1993b, 1994, 1995; Singh and Luis, 1995; Furnham et al., 2004, 2005; Sugiyama, 2004; Andrews et al., 2017). However, many other results on perceived age are inconclusive (Singh, 1993a,b; Henss, 1995, 2000; Singh and Young, 1995; Furnham et al., 1997, 2002, 2005; Sorokowski et al., 2014; Wang et al., 2015). I suggest this is due to two reasons: first, WHR does not have a linear relationship with age (it is U shaped), and secondly, people simultaneously use other cues to infer age. As a consequence, depending on other cues depicted on the stimuli (face, breasts or hair, for example), results can reveal a negative, positive, or null relationship between perceived age and WHR. Further studies investigating the interaction effect between WHR and other physical cues on perceived age are needed. Moreover, the effect of WHR on perceived youthfulness should be explored in different populations, as most of the studies have been conducted in WEIRD countries (but see Furnham et al., 2002; Sugiyama, 2004; Sorokowski et al., 2014 for notable exceptions).

### Cue of Current Pregnancy

According to this hypothesis, a woman's WHR could be used to detect if she is currently pregnant or not. This hypothesis is found in 31% of the papers.

#### Correlation With WHR

Women experience a drastic increase in WHR during pregnancy, which is mainly due to an increase in waist circumference. A decrease in hip circumference may also happen, as fat from this region is mobilized during late pregnancy to meet the needs of the growing fetus (Rebuffé-Scrive et al., 1985; Lassek and Gaulin, 2006). Moreover, unlike for sex and age, WHR is the unique reliable visual cue of pregnancy. The slope of the decreasing attractiveness of WHR through pregnancy for different populations remains to be specified. The earlier in pregnancy the WHR starts to be significantly less attractive, the more plausible this hypothesis will be, as men would be able to use this cue longer/more often.

#### Effect on the Man's Reproductive Success

Because women are infertile while pregnant, pregnancy is directly linked to current fecundity. As such, choosing a pregnant woman as a short-time mate will not enhance a man's reproductive success. However, being pregnant is a transient stage. For a young woman, pregnancy is positively associated with her future expected reproductive success (it is a sign that she is able to become pregnant and to carry a child). However, for older women, being currently pregnant is negatively correlated with the expected number of additional children. Some authors argue that the relationship between current pregnancy and future fertility depends on the fertility rate of the population: if the total fertility rate of the population is low (e.g., two children) it would be costly to be attracted to a woman who is already pregnant, because there is a high risk that she may conceive only one more child. In traditional societies, where total fertility rates sometimes exceed 6, if a woman is pregnant, she may nevertheless conceive at least a few more children (Marlowe and Wetsman, 2001). To sum-up, choosing a mate who is already pregnant will, most of the time, decrease the number of potential descendants of an individual, but the size of the effect depends on the woman's age, the total fertility rate in the population and the type of relationship (short or long term).

Moreover, choosing a long-term mate who is pregnant with the child of another man entails additional evolutionary costs, because investing in a non-biological child decreases the amount of investment an individual can invest in his own descendants. Lastly, Singh suggests that choosing a mate carrying the baby of another man could increase the risks of violence from the jealous current mate (which could impact the survival or future reproductive success of the individual suffering from the attack).

Altogether, mating with a woman with a high WHR because it indicates current pregnancy will have, on average, a negative effect on an individual' reproductive success. Added to the fact that WHR is a reliable and distinctive cue of current pregnancy, this gives solid theoretical support in favor of the present hypothesis.

#### Perception of the Characteristic Using WHR

To my knowledge, only two studies have investigated the role of WHR on the perception of current pregnancy (Furnham et al., 2001; Schützwohl, 2006). The results confirm that a low WHR is associated with a lower perceived probability of current pregnancy. The perception of pregnancy using WHR seems obvious for the last stages of pregnancy, based on profile views or in 3D. However, and even if the conscious awareness of a pregnancy is not mandatory for men's preference to evolve, it would be interesting to explore when exactly people start to detect pregnancy, using 2D images including frontal, back and profile views of women at different pregnancy stages.

### Cue of Parity (Number of Previous Pregnancies)

This hypothesis, first mentioned in 1998 (Yu and Shepard, 1998, see Figure 2), is present in 11% of the papers in this literature. It stipulates that WHR is a way to estimate the number of children (or number of pregnancies) that a woman has previously had in her life.

#### Correlation With WHR

There is evidence that WHR increases with the number of previous pregnancies (independently of age and BMI), due to an increase of waist circumference and/or a relative decrease in hip circumference (Kaye et al., 1990; Smith et al., 1994; Troisi et al., 1995; Bjorkelund et al., 1996; Rodrigues and Costa, 2001; Lassek and Gaulin, 2006; Wells et al., 2010, 2011; Butovskaya et al., 2017). This change in body shape (sometimes referred to as (covert) maternal depletion) is due to the mobilization of fat from the lower parts of the body to meet the needs of the developing child (as well as looser abdominal muscles). This may be interpreted as a life history strategy for allocating energy between competing gluteofemoral fat depots for reproduction, and central fat depots for maintenance and survival (Cashdan, 2008; Wells et al., 2010, 2011). This phenomenon has been observed in various countries: Brazil (Rodrigues and Costa, 2001), Sweden (Bjorkelund et al., 1996), Thailand (Wells et al., 2011), UK (Wells et al., 2010), USA (Kaye et al., 1990; Troisi et al., 1995; Lassek and Gaulin, 2006), and non-industrial societies including tribes from Sub-Saharan Africa, Western Siberia, South America and South Asia (Butovskaya et al., 2017). However, a few other studies find that parity has a negligible or null effect on WHR (Lanska et al., 1985; Tonkelaar et al., 1989; Seidell et al., 1990; Nenko and Jasienska, 2009), but these null results can be explained by the higher average age of the women sampled in those studies. Indeed, the parity effect seems to dissipate over time (Wells et al., 2010). Note that this does not affect the plausibility of the present hypothesis, as the effect of parity on WHR should be visible at the time of mate choice (relatively young).

#### Effect on the Man's Reproductive Success

Women's limited reproductive potential and resources mean that, even controlling for age, each child already born reduces the future number of children a man can sire with the woman if he mates with her long-term (Symons, 1981; Sugiyama, 2005). Parity status influences the survival and quality of future descendants. For example, both high parity and nulliparity are associated with increased risks during childbirth and lower birthweights (Kiely et al., 1986; Fretts et al., 1995; Hinkle et al., 2014; Merklinger-Gruchala et al., 2015, 2017), and IQ is negatively correlated with birth order (Downey, 2001). A recent pregnancy also increases the probability of current infertility because of lactational amenorrhea. Finally, as with current pregnancy, higher parity increases the costs linked to investment in genetically unrelated children.

In conclusion, even when the risks associated with first births are taken into account, choosing a mate with a low parity should have an overall positive impact on individuals' reproductive success (especially for long term relationships), and WHR as a cue of parity is likely to play a significant role in the selection of men's preferences for a low WHR.

#### Perception of the Characteristic Using WHR

To my knowledge, only one study investigates the effect of WHR on perceived parity, with the results validating that women with a higher WHR are perceived as having a higher number of children (Andrews et al., 2017). This study needs replications in populations other than undergraduate students from the USA, but the results suggest that people are using WHR as a cue of parity.

### Cue of Fecundity

One of the most cited argument for an adaptive preference for a low WHR is WHR as a cue of fecundity (cited in 54% of the papers). Healthy women of similar age and reproductive history vary in their ability to become pregnant and achieve a live birth, and WHR would be an indicator of this ability.

#### Correlation With WHR

The most direct evidences in favor of this hypothesis comes from a few clinical studies showing that women with a lower WHR have a higher probability of conception in the case of *in vitro* fertilization and artificial insemination (Zaadstra et al., 1993; Wass et al., 1997). But more recent studies find no relationships between women's WHR and their likelihood of conceiving after induction of ovulation (Imani et al., 2002; Eijkemans et al., 2003). These studies are informative because they are directly linked to fecundity, but women seeking medical assistance to conceive do not represent the ideal population to investigate factors of natural fecundity.

A few studies find that high WHRs are correlated with a later age at first live birth (Kaye et al., 1990) or longer time-to-pregnancy (Wise et al., 2013; McKinnon et al., 2016; but see Wise et al., 2010).

An indirect way to detect the link between fecundity and WHR is to look at the menstrual cycles or at the physiological factors linked to both WHR and fecundity. A few studies indicate that WHR is linked to menstrual abnormalities (Hartz et al., 1984; Moran et al., 1999) and to hormonal levels linked to fecundity (Björntorp, 1991; Jasienska et al., 2004). Similarly, one study finds that women with low WHRs have lower endocervical pH (Jenkins et al., 1995), which helps sperm penetration (Zavos and Cohen, 1980). However, these results seems not to hold for non-obese young women (see Lassek and Gaulin, 2018b for a richer discussion on this topic).

Finally, one study finds that WHR decreases around ovulation (Kirchengast and Gartner, 2002), suggesting that WHR might also reveal whether a woman is at peak cycle fertility. However, these results should be interpreted with caution, as others fail to replicate this effect (Bleske-Rechek et al., 2011).

To conclude, there are some indirect lines of evidence that WHR could be linked to fecundity, but this effect is mostly found when high WHR is associated with other factors (as obesity or older age) and might thus be negligible in populations of young and non-obese women (Lassek and Gaulin, 2018b). Moreover, these studies almost exclusively focus on WEIRD populations, limiting even more the generalization of these results.

#### Effect on the Man's Reproductive Success

Choosing highly fecund mates will increase the reproductive success of a man both for long-term and short-term relationships. In the case of a short-term relationship, it will simply increase the probability of a pregnancy. In the case of a long-term relationship, it will increase the number of potential descendants by reducing both interbirth intervals and the period before the first child (thus increasing the reproductive window).

However, in light of the lack of evidence of a link between WHR and young and non-obese women's fecundity, this hypothesis does not benefit from strong empirical support.

#### Perception of the Characteristic Using WHR

A few studies find that a low WHR is associated with higher perceived fecundity (Singh, 1993b; Furnham et al., 2004; Sugiyama, 2004), but the results are unclear for the vast majority of the cases (Singh, 1993b, 1994, 1995; Singh and Luis, 1995; Furnham et al., 1997, 2001, 2003, 2004, 2005, 2006; Tassinary and Hansen, 1998). I suggest that this lack of clarity is mainly due to the ambiguity of the questions asked to the participants. The main issue is the absence of any indication about the time frame. For example, high parity (linked to a high WHR), is positively associated with past fecundity, but negatively associated with future fecundity [see section Cue of Parity (Number of Previous Pregnancies)]. Thus, in the absence of additional information, it is impossible to know if the participants are rating past, current or future fecundity. The answer probably depends on other cues provided in the survey, or vary from one participant to another, which could explain the inconclusive results. Future tests of perceived fecundity should include the notion of time.

### Cue of Quantity and Availability of “Reproductive Fat”

The idea that fat located around women's hips is qualitatively different from fat found in other body regions, and is used specifically for reproductive functions, exists in the literature since 1993 (Singh, 1993b, see Figure 2). This hypothesis has been progressively enriched, stating that a mother's WHR is linked to the development of her fetus and infant. It is present in 34% of the papers.

#### Correlation With WHR

WHR is, by construction, positively correlated with the quantity of fat situated at the waist level (abdominal fat), and negatively correlated with fat quantity located around the hip (gluteofemoral fat). There is evidence that gluteofemoral fat in women is specific to reproduction: the storing of gluteofemoral fat is high (compared to males and to other body parts) during human female development (Fredriks et al., 2005). Moreover, even with restricted food intake, gluteofemoral fat is metabolically protected from use until late pregnancy and lactation, when it is selectively mobilized (Rebuffé-Scrive et al., 1985; Lassek and Gaulin, 2008). The hypothesis derived from these observations is that the quantity of gluteofemoral fat would have an effect on the development of the fetus during pregnancy and of the infant through lactation.

This reproductive fat appears to be of particular importance for brain development, as gluteofemoral fat is the main source of long-chain polyunsaturated fatty acids that are critical for fetal and infant neural development. Additionally, it seems that abdominal fat inhibits the availability of these neurodevelopment resources (abdominal fat decreases the amount of the enzyme Δ-5 desaturase, which is rate limiting for the synthesis of long-chain polyunsaturated fatty acids; Lassek and Gaulin, 2008). Consequently, WHR is an indicator of the quantity and availability of the fatty acids needed for fetal and infant brain development. In favor of this hypothesis, a study shows that women with lower WHRs and their children have significantly higher cognitive test scores (Lassek and Gaulin, 2008).

Moreover, one study finds that a low WHR correlates with higher birth weight (Pawłowski and Dunbar, 2005), but other studies found the opposite (Brown et al., 1996; Salem et al., 2012).

To conclude, a woman's WHR seems to be a promising indicator of future fetus and infant neural development (although further data from different countries are needed), and additional evidence is required to confirm the link between pre-pregnancy WHR and fetal growth.

#### Effect on the Man's Reproductive Success

Mating with a woman able to provide enough resources during the development of the fetus and infant increases the survival and quality of the descendants. Offspring with higher cognitive abilities are likely to have a better rate of survival and reproductive success than individuals who suffer from worse conditions during their brain development.

A low birthweight is associated with higher infant mortality (Chase, 1969; Behrman et al., 1982; McCormick, 1985) and negative outcomes later in life (Hackman et al., 1983; Baker et al., 2008). However, a low birthweight is also associated with variables which may have no effect on the father's reproductive success (e.g., because occurring late in life), and could even have a positive effect in some environments (Bateson et al., 2004), as a low birthweight seems to be associated with a faster life history strategy (Nettle, 2010).

In conclusion, choosing a mate with a lower WHR if it is linked to higher resources for fetal and infant brain development (and maybe general growth), will have a generally positive impact on a man' reproductive success. However, the size of this effect according to the environmental conditions should be explored. For example, how does this trait impact the number of descendants in the next generation when conditions are more favorable to faster life history strategies?

#### Perception of the Characteristic Using WHR

To my knowledge, only one study explores the effect of WHR on the perceived quality of the descendants (Andrews et al., 2017). Andrews et al. (2017) ask participants to rate female bodies for the following questions: “*If this woman were to have a child, it would be healthy;*” “*If this woman were to have a child, it would make friends easily;*” “*If this woman were to have a child, it would be popular*.” They find a negative relationship between WHR and projected offspring quality, supporting the idea that women with low WHRs are expected to have higher quality children than women with high WHRs (but, as often with this type of questions, it is difficult to tell if we are measuring something else than a halo effect).

### Cue of Health

One of the most cited hypotheses stipulates that a low WHR is an indicator of women's good health (hypothesis present in 87% of the papers). The health conditions which are referred to in the literature on WHR and attractiveness are: cardiovascular diseases, hypertension, strokes, myocardial infarction, diabetes, gallblader disease, kidney diseases, pancreatitis, lung function impairment, cretinism, psychiatric disorder, various cancers and preeclampsia.

#### Correlation With WHR

A high WHR is correlated with many health issues. This claim is supported by abundant evidence (for reviews see Björntorp, 1987a,b, 1993; Manolopoulos et al., 2010). However, these findings are based on relatively old women or men (often 60 years old or more, almost never before 30), mostly suffering from some degree of obesity, raising the possibility that this relationship is not present for evolutionary relevant reproductive-age populations (Lassek and Gaulin, 2018a).

#### Effect on the Man's Reproductive Success

The consequences for reproductive success of mating with a woman with a low WHR because it is a cue of her health are not straightforward. First, the cited health conditions are not contagious, thus the survival of the woman's mate cannot be directly affected. Secondly, most of the chronic diseases associated with WHR are recent, from an evolutionary point of view, and they are associated with present-day environments, lifestyle and alimentation (Eaton and Eaton, 1997; Groop, 2000). Third, even if we assume that these health issues were common in our evolutionary past, most of them appear late in life, after the end of women's reproductive life. Thus, most of the heath issues linked to high WHRs are unlikely to affect the number of descendants of a woman's mate (Lassek and Gaulin, 2018a).

A few exceptions in the list of WHR-related health issues can be made, however. First, a high WHR early in pregnancy seems to be correlated to higher risks of preeclampsia (a condition which can be fatal to both the fetus and mother; Yamamoto et al., 2001; Taebi et al., 2015). However, evidence is needed to see if preeclampsia is predicted by WHR *before* pregnancy (when mate choice occurs). One paper indicates that a high WHR can be an indicator of cretinism (a syndrome often linked to infertility; Streeter and McBurney, 2003). However, WHR is probably not a very good cue to detect cretinism, as this health condition generates other physical modifications, more easily noticeable than WHR (Chen and Hetzel, 2010). Another exception is the polycystic ovarian syndrome. This condition can affect the fertility of young women, but only when the syndrome is associated with obesity (Pall et al., 2006; Pasquali et al., 2006). And again, the prevalence of this condition in our evolutionary past is unclear. Lastly, the term “health” can include malnutrition and parasites (although it is almost never referred to in the literature), which can affect fertility at any age and are not restricted to our contemporary societies. These two last characteristics are discussed in the next sections of this paper (Cue of Parasite Load & Cue of Diet).

Health later in life could influence the survival and quality of descendants in another way, through maternal investment: long-term health and longevity increase the probability of having a living and healthy mother able to provide care for children and grandchildren (Sear et al., 2000). Thus, theoretically, WHR as a cue of health could have played a role in the selection of preferences for a low WHR. However, this hypothesis holds only if WHR at a younger age (at the time of mate choice) is a reliable predictor of health later in life, excluding diseases which are evolutionary novel. Longitudinal studies in non-WEIRD populations are needed to explore this possibility.

Alternatively, good health at old age could be related to genetic quality. Descendants from individuals with higher longevity could have a better health, even at younger ages. In this case, men's preferences for a low WHR as a cue to health could evolve through indirect selection. Cross-generational studies are needed to test this good genes hypothesis.

To conclude, in the light of the present evidence, the “WHR as a cue of health” hypothesis is unlikely to be at the evolutionary origins of preferences for a low WHR in young women. However, this hypothesis could receive new theoretical support through the maternal and grandmaternal investment or the genetic quality hypotheses, but only if some of the above predictions (links between women's WHR at young age and health at old age, or health of the descendants, excluding evolutionary novel diseases) are supported by evidence.

#### Perception of the Characteristic Using WHR

Participants are asked to rate the health of the stimuli in many studies (Singh, 1993a,b, 1994, 1995; Singh and Luis, 1995; Singh and Young, 1995; Furnham et al., 1997, 1998, 2001, 2002, 2003, 2004, 2005, 2006; Yu and Shepard, 1998; Wetsman and Marlowe, 1999; Henss, 2000; Marlowe and Wetsman, 2001; Sugiyama, 2004; Marlowe et al., 2005; Schützwohl, 2006; Tovée et al., 2007; Swami et al., 2009; Sorokowski et al., 2014). In general, a low WHR is associated with better perceived health. Interestingly, however, a few studies investigating non-WEIRD populations find a null or positive effect of WHR on perceived health (Yu and Shepard, 1998; Wetsman and Marlowe, 1999; Tovée et al., 2007; Sorokowski et al., 2014). This support the idea that the association between high WHR and poor health might be valid in contemporary western countries only. Even if, as explained earlier, the perception of health using WHR is not a mandatory step to validate the hypothesis, more research (with different stimuli and questions) is needed to clarify this point.

It would also be interesting to see if young women's WHR is linked to their perceived future health and longevity. One could also explore if individuals have any idea of the kind of diseases associated with WHR.

To my knowledge, only one study explores the effect of WHR on the perceived quality of the descendants (Andrews et al., 2017, see section Cue of Quantity and Availability of “Reproductive Fat” above). They find a negative relationship between women's WHR and the projected offspring quality, in accordance with the hypothesis of WHR as a cue of genetic quality.

### Cue of Parasite Load

The idea that WHR could be a sign of infection by parasites is not recent (e.g., Furnham et al., 1998, see Figure 2) but is quite rare in the literature (in 5% of the papers).

#### Correlation With WHR

Some parasites, including intestinal worms, can increase waist size through oedema while causing weight loss, which will result in a higher WHR (Cross, 1992; Kucik et al., 2004).

#### Effect on the Man's Reproductive Success

Parasite load can affect survival and fertility. Moreover, most parasites are contagious, and mating with a woman carrying parasites increases the probability of being infected. As such, WHR as a cue of parasite load can have an effect on a man's health and survival, as well as an effect on the number, survival and quality of descendants he can sire with the infected woman. This effect remains to be quantified and will certainly vary according to the frequencies and types of parasites present in the environment.

WHR as a cue of parasite load is an interesting hypothesis, but it has been largely overlooked and evidence is by consequence lacking.

#### Perception of the Characteristic Using WHR

There is no specific research on the perception of parasite load based on WHRs. However, many studies explore the effect of WHR on perceived general health (see section Cue of Health).

### Cue of Diet or Malnutrition

The hypothesis that WHR could be a cue of women's diet or malnutrition is found in 5% of the papers.

#### Correlation With WHR

One paper mentions that a high WHR could be a sign of Kwashiorkor, a form of malnutrition (Streeter and McBurney, 2003). Indeed, WHR can increases in some cases of malnutrition because of the presence of an oedema enlarging waist size (Golden, 1982; Waterlow, 1984).

A diet rich in fibrous food can also increases waist size and thus WHR. For example, Marlowe states that Hadza women may have a high WHR because “a larger gut is required to hold the amount of bulky, fibrous tubers in the Hadza diet” (Marlowe et al., 2005).

#### Effect on the Man's Reproductive Success

Malnutrition increases the morbidity and mortality of a woman and her children, and might also decreases her fecundity (Mosley, 1977; Osteria, 1982; Hernández-Julián et al., 2014). Choosing a mate suffering from malnutrition will thus decrease one's reproductive success. The prevalence of malnutrition involving a high WHR during our evolutionary past should be explored, to establish if it could have represented an evolutionary force for the preferences toward low WHRs.

Concerning diet, it is not clear if a large waist reveals a good ability to digest fibrous food or a poor ability to assimilate this kind of food. If the latter is true, a higher WHR will be associated with less resources available for pregnancy and lactation, leading to lower survival and quality of descendants. The opposite will be true if a large waist is associated with a better ability to digest fibrous food.

The hypotheses of WHR as a cue of malnutrition or diet (or ability to digest some type of food) have been mainly ignored, and evidence is thus missing.

#### Perception of the Characteristic Using WHR

There is no specific research on the perception of diet or malnutrition based on WHR.

### Cue of Fetal Conditions

This hypothesis is mentioned only once in the literature (Singh, 1995). It stipulates that the WHR of an adult woman could be an indication of her developmental conditions before her birth.

#### Correlation With WHR

A negative link between adult WHR and birth weight, or placental weight to birth weight ratio (an indicator of retarded fetal growth), has been found, but this study is only composed of men over 50 years old (Law et al., 1992). To my knowledge, there is no empirical evidence showing that young women's WHR is a reliable cue of their fetal development.

#### Effect on the Man's Reproductive Success

A low birthweight is associated with higher infant mortality (Chase, 1969; Behrman et al., 1982), but this cannot affect a mate's reproductive success, as the mating occurs after the woman's survival to infancy. But a low birthweight also has some negative outcomes later in life (Bateson et al., 2004), for women's fertility (Hackman et al., 1983) and longevity (Baker et al., 2008, which decreases the likelihood of having a living and healthy mother caring for her mate's descendants, see section Cue of Health).

On the other hand, as explained in section Cue of Quantity and Availability of “Reproductive Fat,” a low birthweight is also associated with some advantages in harsh environments (Bateson et al., 2004), as well as a relatively early sexual maturation and reproduction (Nettle, 2010), which might increase the number of descendants for the potential mate.

To conclude, WHR as a cue of a woman's fetal condition could have, in theory, a negative, positive or null effect on her mate's reproductive success. Combined with the fact that the link between WHR and fetal conditions has been shown for older men only, this hypothesis lacks both empirical and theoretical support.

#### Perception of the Characteristic Using WHR

There is no test of the effect of WHR on perceived fetal conditions.

### Cue of Pelvis Size

This hypothesis, found in 16% of the papers in this literature, is already reported in one of the first papers from Singh (1993b), see Figure 2), and states that WHR is a cue of the size (or shape) of women's pelvis.

#### Correlation With WHR

WHR is, by definition, linked to hip size, which is indicative of underlying pelvic skeletal morphology. It is unclear, however, how much of the variation in WHR is explained by pelvic size (it seems that most of the variance in WHR is due to fat storage on the hip and waist regions).

#### Effect on the Man's Reproductive Success

The size of the pelvis determines the size of the bony pelvic canal through which the fetus passes during a delivery. As such, a wider pelvis reduces the risk of obstructed labor (Caldwell and Moloy, 1933; Stålberg et al., 2006). In the absence of healthcare, women who are unable to deliver their babies perish, along with their babies. Moreover, obstructed labor can lead to many long-term health issues on both sides, which can influence future survival and fertility. Thus, a woman's small pelvis will decrease the number of descendants a man can sire with her.

However, a large pelvis can be an obstacle to efficient locomotion (Leutenegger, 1974; Lovejoy, 1988; Ruff, 2017 but see Warrener et al., 2015). A woman with a lower ability to walk will have higher difficulties to secure resources for her children, which will decrease their survival or quality. Altogether, stabilizing selection is expected to be operating on female hip size, as well as on men's preferences for this trait.

To conclude, the evolutionary costs and benefits of a wide pelvis seem more appropriate to explain the origin of the sexually dimorphic hip size via natural selection, than to explain men's preferences for a specific WHR. Female pelvic size and shape are the result of two conflicting evolutionary pressures: bipedal locomotion and parturition of a highly encephalized fetus (Leutenegger, 1974; Lovejoy, 1988; Rosenberg and Trevathan, 1995 but see Leong, 2006; Betti and Manica, 2018). It is possible that the link between pelvic size and childbirth and locomotion contributed to the selection of men's preference for an average hip size, but more research is needed to confirm its effect on men's reproductive success.

#### Perception of the Characteristic Using WHR

To my knowledge, nobody has tested the effect of WHR on perceived difficulties during childbirth, or on perceived locomotion.

### Cue of Center of Body Mass

This hypothesis, suggested by Pawlowski and Dunbar (2001) and Pawłowski and Grabarczyk (2003) and found in 6% of the papers in the literature, stipulates that WHR is linked to the position of the body's center of gravity, which influences bipedal stability.

#### Correlation With WHR

Everything else being equal, a lower WHR will lower the center of mass of the body. One study uses body measurements of young women to experimentally establish the correlation between WHR and the center of body mass (Pawłowski and Grabarczyk, 2003). However, the correlation is not very strong in their sample of students, and more data is required.

#### Effect on the Man's Reproductive Success

In advanced pregnancy and during lactation, when the infant is being carried, a bipedal female has to contend with a substantial increase in the anterior load above the center of gravity (Pawłowski, 2001). Fat deposits in the buttocks and thighs may prevent the center of gravity from moving upwards and forwards, and facilitate walking and foraging during pregnancy and lactation. Choosing a mate with a lower center of gravity could increase the survival of the fetus and infants a man would sire with this woman, as she would be less likely to fall and injure the fetus, the infant or herself, and she would be more successful in foraging or escaping predation during these critical periods. A lower center of gravity would also mean a lower energetic cost to maintain balance, and thus an increase in resources available to be directed toward the descendants. Thus, a woman's center of gravity could have an effect on her mate's reproductive success (Pawlowski and Dunbar, 2001; Pawlowski, 2003).

However, as with the pelvic size argument, this hypothesis seems more suitable to explain the origin of dimorphic body shapes in the human species than to explain men's preferences.

#### Perception of the Characteristic Using WHR

To my knowledge, there has been no research concerning WHR and perceived center of body mass, or perceived walking abilities during pregnancy and lactation.

### Cue of Ability to Cope With Stress

The link between stress and women's WHR exists in the literature since 1995 (Singh, 1995, see Figure 2), but is included in only 5% of the papers. Depending on the author, a high WHR could be a sign of exogenous stress, a cue of a poor ability to cope with stress, or a cue of an effective response to stress.

#### Correlation With WHR

Compared to women with low WHRs, women with high WHRs report more chronic stress and have more psychological and psychiatric issues (Björntorp, 1987b, 1993). According to Björntorp, a high WHR might be interpreted as a sign of an inability to cope with environmental stress. One experiment shows that women with high WHRs evaluate laboratory challenges as more threatening, performed more poorly on them, and reported more chronic stress (Epel et al., 2000).

However, Cashdan draws an opposite conclusion from the same observations (Cashdan, 2008). Cortisol (the levels of which are associated with WHR) enables the mind and body to respond effectively to stress, by shifting energy substrates from storage sites to the bloodstream and by increasing blood pressure and cardiac output. As part of this response, cortisol increases WHR by increasing visceral fat. Conversely, stress-induced cortisol secretion is greater among women with more central fat (Epel et al., 2000).

To conclude, WHR seems to be related to stress responses, but it is not clear if a low WHR is a cue of a good or a poor ability to cope with environmental stress. The stress responses in women with high WHRs may be maladaptive in most WEIRD populations, yet it could be adaptive where conditions are extreme or where stress is episodic rather than constant (Cashdan, 2008).

#### Effect on the Man's Reproductive Success

If a high WHR is a sign of inadequate coping with stress, women with a high WHR may bear descendants of lower quality because they may be less able to secure resources or provide care for them. However, the opposite is true if a high WHR is a sign of a better ability to respond to stress.

Maternal stress during fetal growth can lead to a lower birthweight. Stress also has epigenetic effects on offspring' life history trajectories and health (Worthman and Kuzara, 2005). However, according to the adaptationist life history perspective, these effects could be associated with a phenotype adapted to the environment (Bateson et al., 2004; Worthman and Kuzara, 2005; Nettle, 2010, see section Cue of Quantity and Availability of “Reproductive Fat”).

To conclude, it is unclear if choosing a woman with a lower WHR, as a cue of stress responses, would have a positive, neutral or negative impact on a man's reproductive success. The answer will probably differ according to the environment, and could lead to a preference for a relatively high WHR in some cases (Cashdan, 2008).

Overall, this hypothesis lacks clarity. Nevertheless, the link between stress and WHR is a valuable explanation of the variability of women's WHRs (Cashdan, 2008).

#### Perception of the Characteristic Using WHR

To my knowledge, the effect of WHR on perceived stress, or ability to cope with stress, has not been investigated.

### Cue of Ability to Acquire Resources

It has been suggested that a preference for a relatively high WHR could be adaptive in some environments because the hormonal profile associated with high WHRs (high androgen and cortisol, low estrogen) may favor success in resource competition, particularly under stressful and difficult circumstances (Cashdan, 2008). This hypothesis is mentioned in 10% of the papers.

#### Correlation With WHR

High androgen levels in women are associated both with higher WHR (Evans et al., 1983; Elbers et al., 1997; Santoro et al., 2005; van Anders and Hampson, 2005) and with greater assertiveness, competitiveness and aggressiveness in women (Purifoy and Koopmans, 1979; Baucom et al., 1985; Dabbs et al., 1988; Cashdan, 1995; Udry et al., 1995; Harris et al., 1996; Dabbs and Hargrove, 1997; Grant and France, 2001; von der Pahlen et al., 2002). Androgens also increase muscle mass and physical strength (Bhasin et al., 1996). Unfortunately, these studies have been conducted in western countries only, limiting the generalization of the results to other populations.

Androgens also shape features other than WHR (including facial traits, body features and voice; Abitbol et al., 1999; Rickenlund et al., 2003; Lefevre et al., 2013; Whitehouse et al., 2015), and individuals can rely on other cues to assess androgen levels. More importantly, men could use more direct cues of the ability to access resources (e.g., behavior, physical accomplishments or quantity of resources) and may not need indirect cues.

#### Effect on the Man's Reproductive Success

According to this hypothesis, having a relatively high WHR can increase a woman's survival and reproductive success, because she will be more able to work hard to support herself and her children, compete directly for resources for them, and cope with resource scarcity. Most of these effects will translate into positive effects on her mate's reproductive success.

In this case, the optimal female WHR (for herself and her mate) is likely to vary with the circumstances. In societies where women are expected to provide most of the food, through hard physical work and competition, the balance should be tipped toward a hormonal profile consistent with a higher WHR. In more benign conditions, where women get most of their resources from investing men, a hormonal profile consistent with a low WHR might be more adaptive (Cashdan, 2008).

Overall, as proposed by Cashdan herself, this hypothesis is more likely to explain the variations in women's WHRs (between environments and within lifetime) than to account for men's preferences (Cashdan, 2008). However, we cannot exclude that the link between WHR and women's ability to acquire resources might play a role in the variations observed in the exact value of the preferred WHR between populations.

#### Perception of the Characteristic Using WHR

One study investigating perceived aggressiveness finds no effect of WHR (Singh, 1994). Another study finds no effect of WHR on factors linked to perceived ambition, independence, self-confidence and success (Henss, 1995). Two studies find that figures with low WHRs are rated as more dominant than figures with high WHRs (Henss, 2000; Buunk and Dijkstra, 2005), which goes in the opposite direction of what is expected according to the present hypothesis. However, these studies are designed to investigate the competition for a mate, not the competition for resources. Studies exploring the effect of WHR on the perceived ability to acquire resources (and not mates) are needed.

### Cue of Sex Ratio and Level of Testosterone in Descendants

This hypothesis includes two different sub-hypotheses. The first one, suggested by Manning et al. (1996), stipulates that women with a high WHR have more sons than women with a low WHR, controlling for the total number of children. The second hypothesis states that women with a high WHR have children exhibiting higher levels of testosterone. Pooled together, these two hypotheses are found in 4% of the literature (see Figure 2).

#### Correlation With WHR

A few studies show that a woman's WHR is positively correlated with her number of sons (Manning et al., 1996, 1999; Singh and Zambarano, 1997). However, these studies are measuring women who already have children and correlate WHR with the proportion of existing sons, and it is possible that having sons results in a greater increase in WHR than does having daughters. A more recent study looking at pre-conception WHR and offspring gender finds no significant correlation (Tovée et al., 2001). Thus, there is not enough evidence supporting the fact that a high WHR would be related to more sons in the future.

Manning also found that women with high WHRs tended to have children with low 2D:4D ratios (Manning et al., 1999). A low 2D:4D ratio is supposed to be correlated with high testosterone levels, and the authors conclude that women with high WHRs have more masculine children. However, there is new evidence that the 2D:4D is not a reliable indicator of the levels of testosterone (Hollier et al., 2015; Whitehouse et al., 2015; Apicella et al., 2016).

In conclusion, the idea that a woman with a high WHR will produce more sons or more masculine children is not supported by empirical data.

#### Effect on the Man's Reproductive Success

Several theories postulate that the sex of the descendants can influence an individual's reproductive success (Hiraiwa-Hasegawa, 1993; Hiraishi et al., 2016). The advantage of sons over daughters depends on various characteristics of both the parents (condition or rank) and the population (including dispersal patterns, inheritance of rank or resources, and degrees of local resource competition). In some cases, one sex has a greater chance of survival and a higher potential reproductive success than the other.

In the hypothetical case where high-WHR women would have children with high testosterone levels, choosing a mate with a relatively high WHR could represent an advantage in some environments. High testosterone is related to various characteristics (from muscular strength to competitive behavior; Bhasin et al., 1996; Apicella et al., 2011, 2015; Schipper, 2014), which could lead to a higher survival and a higher reproductive success.

To conclude, choosing a mate likely to produce more sons or more masculine children could increase the reproductive success of an individual, but it will depend on the environment and on the of the individuals' condition. More importantly, there is no solid evidence that WHR is an indicator of the sex ratio or masculinity of the future descendants. This hypothesis is therefore not supported by empirical evidences.

#### Perception of the Characteristic Using WHR

The effect of women's WHR on the perception of their children's sex ratio or masculinity has never been investigated.

### Cue of Sexual or Maternal Behavior

Interestingly, the idea that a woman's WHR is linked to her behavior and personality, as perceived by others, is found in many of the pioneering papers of this literature (Singh, 1993a,b, 1994; Henss, 1995; Singh and Luis, 1995; Singh and Young, 1995; Furnham et al., 1998, 2004, 2005; Sugiyama, 2004). However, clear mentions of the hypothesis that WHR could be used as a predictor of past and future behavior by men to choose a mate are rare (2% of the papers) and recent (see Figure 2).

#### Correlation With WHR

Compared to women with a high WHR, women with a low WHR tend to have a less restricted sociosexuality, sexual intercourse at an earlier age, more sexual partners, and more extrapair copulations (Mikach and Bailey, 1999; Hughes and Gallup, 2003; Fisher et al., 2016). The question remains whether this correlation is due to different preferences and behaviors expressed by women (with hormonal levels as a potential proximal mechanism), or if it only reflects the different opportunities linked to different levels of physical attractiveness. In the latter case, this correlation cannot explain the origin of male preferences for a certain WHR [but it could potentially explain its maintenance, see section Cue of Sexy Daughters (Fisherian Runaway Model)].

Estrogen, testosterone and cortisol levels, all influencing WHR, are linked to maternal investment in many species, including humans (Fleming et al., 1997; Bardi et al., 2001; Dwyer et al., 2004). Thus, WHR could be a cue of women's maternal tendencies. However, there is no direct evidence of a correlation between a woman's WHR before pregnancy and her future maternal investment. Only a few studies provide some indirect evidences for this hypothesis, by showing a correlation between hormonal levels and reported maternal tendencies (Deady and Law Smith, 2006; Deady et al., 2006; Law Smith et al., 2012).

To conclude, more direct evidence is needed to validate the links and mechanisms between women's WHR and their behavior.

#### Effect on the Man's Reproductive Success

The effect of women's sexual behavior on their mates' reproductive success is double-edged. Women with unrestricted sociosexual orientations, relative to those with more restricted orientations, are more likely to engage in sex at an earlier point in their relationships and have more sexual partners (Simpson and Gangestad, 1991). Thus, being attracted to women with a less restricted sociosexuality might increase the man's chances of mating. On the other hand, women with unrestricted sociosexuality are also more willing to engage in and report higher levels of extradyadic activity (Seal et al., 1994; Barta and Kiene, 2005; Rodrigues et al., 2017; Weiser et al., 2018), therefore increasing the risk of extra-pair copulation costs for their mate (see section Cue of Current Pregnancy). However, these results need to be replicated in non-WEIRD populations before drawing any strong conclusions.

Mating with a woman with a less restricted sociosexuality also increases the risks of being contaminated by sexually transmitted diseases (Hall, 2012). Women with unrestricted sociosexual orientations report more casual sex encounters and multiple and concurrent sexual partners, factors known to increase the risk for exposure to sexually transmitted diseases (Seal and Agostinelli, 1994; Hoyle et al., 2000).

In sum, the effects of a less restricted sociosexuality on the mate's reproductive success are potentially positive for a short-term relationship if the occurrence of sexually transmitted diseases in the population is low, and probably null or negative for a long-term relationship.

Higher maternal investment can increase the survival and quality of the descendants. However, as stated earlier, to this date there is no direct empirical evidence supporting pre-pregnancy WHR as a cue of future maternal investment.

Overall, this hypothesis has not been explored in many papers, and lacks empirical and theoretical support.

#### Perception of the Characteristic Using WHR

Several of the early studies investigate the effect of WHR on perceived behavioral and personality traits, but these papers do not include any theoretical background regarding WHR as a potential cue of behavior or personality (Singh, 1993a,b, 1994; Henss, 1995; Singh and Luis, 1995; Singh and Young, 1995; Furnham et al., 1998, 2004, 2005; Sugiyama, 2004). The absence of prediction in these papers is problematic, as the questions asked to the participants are sometimes unclear, and the authors often pooled together items which are linked to different hypotheses, making it impossible to properly test the hypothesis.

Some authors explore the effect of WHR on perceived traits like “*desire for children*,” “*likes children*,” “*good parent*,” or “*nurturing*” (Singh, 1993a,b, 1994; Henss, 1995; Singh and Luis, 1995; Furnham et al., 2005), but the results are inconsistent. Thus, there is no good evidence that WHR is perceived as a cue of maternal behavior, but more appropriate tests with clear predictions are needed.

In a few studies, participants rated figures with high WHRs as more “*faithful*” (Singh, 1994; Singh and Young, 1995). Other studies find that figures with a low WHR are perceived as more “*flirtatious*” (Furnham et al., 2005). These results are in accordance with the hypothesis that WHR serves as a cue of sexual behavior.

### Cue of Sexy Daughters (Fisherian Runaway Model)

Fisher famously described a process whereby a small initial preference ultimately leads to extreme traits and preferences through “runaway” selection (Fisher, 1930). If a particular trait in one sex is preferred in mates, then genes disposing stronger preference for the trait could spread as they become linked with genes predisposing the preferred trait.

This hypothesis is not specific to WHR. In fact, the runaway process is almost never applied to men's preferences for WHR. Yet, in one paper, Singh explains that WHR is heritable and “*offspring of women with lower, more feminine, WHR would have inherited good health and would have been physically attractive to potential mates*” (Singh and Randall, 2007). Tassinary also refers to the runaway model, especially to explain why very small WHRs could theoretically be attractive to men (Tassinary and Hansen, 1998).

#### Correlation With WHR

For this hypothesis to be valid, WHR needs to be genetically heritable, and there is some evidence that this is the case (Donahue et al., 1992; Bouchard et al., 1996; Schousboe et al., 2004). According to this hypothesis, daughters of women with a low WHR will have a lower WHR and thus will be more attractive. The hypothesis also requires some heritability of preferences for a low WHR. However, this heritability may cease to be observed once the preference invades the population (since there will not be enough variance in the preferences left). Importantly, this hypothesis does not require any link between WHR and any physiological quality.

#### Effect on the Man's Reproductive Success

According to this hypothesis, a man mating with a woman with a low WHR will have more attractive daughters than if he mates with a woman with a high WHR. These attractive daughters will have a higher mating and thus reproductive success in the next generation in a population with men attracted by low WHRs, which will have a positive impact on their father's reproductive success. The size of the effect of women's WHR on their daughters' reproductive success remains to be identified. Indirect evidence can be found in studies showing that a low WHR is linked to a higher number of sexual partners, as a proxy for mating success (Mikach and Bailey, 1999; Hughes and Gallup, 2003).

It is important to point out that this hypothesis slightly differs from the other ones in this review because it only involves indirect selection on men's preferences. A man's mating preference is favored by direct selection if it increases his own lifetime reproductive output, and by indirect selection if his preference increases the reproductive output of his offspring. Some authors have shown that indirect selection on mate choice via the sexual attractiveness of offspring is a weak evolutionary force relative to direct selection (Kirkpatrick and Barton, 1997). However, such statements of relative strength should not be taken to imply that indirect selection is of little evolutionary importance (Kokko et al., 2003). This would be true only if direct and indirect selections were opposed, which does not seem to be the case for men's preference for WHR (most of the hypotheses point toward a preference for a low WHR). This hypothesis can then be seen as an additional force reinforcing direct selection on men's preferences.

Another possible limitation regarding this hypothesis is the indirect cost of sexual antagonism. If WHR is genetically heritable for both sexes, men will have to trade off higher sexiness in daughters with lower-quality sons when choosing a mate, as optimal WHR value differ between men and women (Rice and Chippindale, 2001). Measures of the heritability of WHR for both sexes is necessary to determine the existence of this indirect cost.

#### Perception of the Characteristic Using WHR

To my knowledge, there is no study investigating the effect of WHR on perceived attractiveness of a woman's future descendants. The only questions somehow related to this issue are asked by Andrews et al. (2017): “*If this woman were to have a child, it would make friends easily;*” “*If this woman were to have a child, it would be popular*.” They find that the ratings for these items are higher for women with low WHRs. However, these questions were not specifically designed to explore this particular hypothesis.

### Summary of Hypotheses Plausibility

The conclusions of the theoretical analyses of each hypothesis presented in this paper are summarized in Table 1. This classification is obviously not definitive and is anticipated to change according to the discovery of new evidence.

**Table 1:** Proposition of classification of the hypotheses found in the literature, according to their theoretical plausibility.

	**Unfit hypothesis in light of the current evidence**	**More evidence is needed to evaluate the plausibility of the hypothesis**	**Plausible hypothesis for the emergence of men's preferences**	**Plausible hypothesis for the maintenance of men's preferences**	**Plausible hypothesis for the selection of women's WHR**
1. Cue of biological sex			X	X	
2. Cue of (reproductive) age			X	X	
3. Cue of current pregnancy			X	X	
4. Cue of parity			X	X	
5. Cue of fecundity		X			
6. Cue of quantity and availability of “reproductive fat”			X	X	
7. Cue of health		X			
8. Cue of parasite load		X			
9. Cue of diet		X			
10. Cue of fetal conditions	X				
11. Cue of pelvis size		X			X
12. Cue of center of body mass					X
13. Cue of ability to cope with stress		X			
14. Cue of ability to acquire resources				X	X
15. Cue of sex ratio and level of testosterone in descendants	X				
16. Cue of sexual or maternal behavior		X			
17. Cue of sexy daughters (Fisherian runaway model)				X	

*Some hypotheses are unlikely to explain men's preferences toward a certain WHR (column 1). Some hypotheses are likely to explain the emergence of men's preferences during our evolutionary history (column 3), while others better explain the maintenance of these preferences, once they already emerged in the population (column 4). Some hypotheses are good explanations for the selection of a certain WHR in human populations, but do not necessarily lead to a selection of men's preferences (column 5). Finally, some hypotheses need further investigation before one could properly estimate their plausibility (column 2). Both categories and hypotheses are non-exclusive*.

## Discussion

In this paper, I review the hypotheses explaining why men's preferences for a certain WHR in women may have been selected in the human species. These hypotheses are numerous, and overall, there is some solid theoretical and empirical support in favor of a selection of men's preferences for a mate with a relatively low WHR (with some variations on the exact value according to the population and the environment). However, many of the papers on this topic do not properly develop the theoretical framework, and some interesting hypotheses have been overlooked, while some of the most popular hypotheses require stronger theoretical or empirical support.

To show that men's preference for a certain WHR is an adaptation, it is necessary to demonstrate that a man choosing a mate with a certain WHR will benefit from an increase in reproductive success. Thus, it is crucial to describe the consequences of the preference and show that it can have an impact on the quantity or quality of men's descendants. Importantly, the ultimate focus here is the reproductive success of the individual who is expressing the preference, not of the woman displaying a certain WHR.

WHR as a cue of women's health is one of the most cited hypotheses, appearing in 87% of the papers examined in this review, although health issues linked to WHR have a very limited impact on the women's mates' reproductive success. WHR as a cue of women's fecundity is a notorious hypothesis but is not supported by empirical evidence among populations of young and non-obese women (which is the population of interest for the hypothesis). On the other hand, two hypotheses which are particularly good candidates (WHR as a cue of current pregnancy and parity) are too often forgotten in the literature. Some hypotheses are promising but have been largely overlooked (e.g., WHR as a cue of parasite load, diet or “sexy daughters”). WHR as a cue of quantity and availability of “reproductive fat” hypothesis has received decent empirical and theoretical support and is now generally accepted in the field. WHR as a cue of sex ratio and levels of testosterone in descendants is not supported by empirical evidence, and has therefore never taken hold in the field. Other interesting hypotheses are better suited to explain the presence of a sexually dimorphic WHR in our species through natural selection than men's preferences: WHR as a cue of pelvis size and center of body mass. The preference for slightly higher WHRs in some populations can be explain by WHR as a cue of the ability to acquire resources, although this hypothesis is primarily an excellent account for the variability of women's WHRs. Crucially, the numerous hypotheses reviewed in this paper are not mutually exclusive. The most likely scenario incorporates several of these hypotheses, operating at different periods of our evolutionary history.

To summarize, WHR is a powerful measure (as shown by the numerous physical and physiological characteristics correlated with it), but it may not be as “magical” as often assumed, and not all the features correlated with WHR are linked to mate value. Most of the mate value-related information provided by WHR is relatively basic (sex, age, number of children, current pregnancy). Nevertheless, WHR is a useful and practical visual trait aggregating the information that a potential mate might not even known is associated with an increase in his own reproductive success.

Non-adaptive explanations for men's preferences toward a certain WHR are not the focus of this paper but they are not necessarily refuted. For example, some authors argue that low WHR preferences may be the result of a generic psychological mechanism of enhanced responding to exaggerated features, or “supernormal” stimuli (Gray et al., 2003). According to this hypothesis, if men view a low WHR as “typical” of female bodies, this could lead men to prefer female WHRs that are even lower than normally attainable (Gray et al., 2003). However, this hypothesis still requires that men use WHR as a cue of biological sex (a hypothesis reviewed in this paper). Men's preferences for a certain WHR can also be explained by sociocultural theories. For example, it is argued that cross-cultural variations in men's preferences for women's WHR could be based on the gender roles occupied by men and women in different cultural settings (Swami et al., 2006a,b). But this hypothesis still requires an explanation regarding the origin of the association between WHR and a certain gender role. Finally, as mentioned earlier, some authors have argued that WHR might not be the best cue of a woman's mate value and that its correlation with attractiveness might be an artifact of men's preferences for another physical characteristic (Tassinary and Hansen, 1998; Tovée et al., 1999; Furnham et al., 2005; Cornelissen et al., 2009a,b; Brooks et al., 2010, 2015; Lassek and Gaulin, 2016). A similar systematic review focusing on a different measure instead of WHR might thus reveal a different picture than the one depicted here (although a few hypotheses concerning men's preferences for features correlated with WHR are incidentally already included in the present review).

The sketch presented by this review calls for more theoretical rigor and precision (and, to be clear, I do include myself in this criticism). Confusion about the theoretical framework can lead to inadequate predictions and suboptimal experimental designs. For example, the stimuli created to test the “WHR as a cue of current pregnancy” hypothesis should be different from the one used to test the “WHR as a cue of (reproductive) age,” in terms of WHR range, WHR manipulation (hip or waist changes), and associations with other visual cues (e.g., age of the face). The questions asked to participants to explore the perception of characteristics induced by WHR are often too vague or inadequate, perhaps due to ambiguity in the underlying predictions. The imprecision of the predictions tested previously may have contributed to the increasing number of studies that find null results when testing evolutionary hypotheses for human mating preferences. Null results are not an issue *per se*, but the repeated failure to validate unsound predictions may incorrectly lead to the rejection of an evolutionary explanation to human mate preferences, thus undermining well-founded hypotheses by discrediting the general research paradigm. Finally, the posited theoretical framework will inherently drive the search for the empirical evidence necessary to support a hypothesis. Thus, it is possible that some of the hypotheses presented here would have received more empirical evidences if the theory had been clearer. For example, most of the evidence used to support the “WHR as a cue of health” hypothesis is not theoretically relevant (health issues at old age or evolutionary recent diseases), maybe in part because of an underspecified theory. With new and more precise predictions, as outlined in this review, additional evidences could be discovered through a deeper exploration of the relevant literatures.

This review has several limitations and should be regarded as a first step to a deeper understanding of this research question, and as a source of ideas to further test the evolutionary origins of mate preferences. I focus only on published research, as the aim is to inventory hypotheses accepted by the academic community, as well as their recognized justifications. However, an examination of unpublished data would be an important next step, in particular to give additional empirical support for the (im)plausibility of the different hypotheses. Moreover, the tentative classification of the different hypotheses presented in Table 1 is based on their examination through verbal theorizing, but formal models might be helpful to provide a more objective way to define the likelihood of the different scenarios. Quantitative data on the correlations between WHR and the hypothesized traits needs to be gathered, as it would help for the specification of the parameters in such models. Additional layers could be explored to further scrutinize the plausibility of the hypotheses. For example, scenarios where a positive link between women's WHR and her offspring's reproductive success is a necessary condition would require a stronger selection than scenarios based on a higher number and survival of offspring.

In this review, I focus on the literature regarding men's adaptive preferences for women's WHR, but the criticism presented here could be applied to other research topics in evolutionary psychology. It is crucial to establish the evolutionary plausibility of existing hypotheses. Otherwise, we risk hanging on too long to implausible—although often parsimonious—explanations, which can harm the credibility of our field in the long run. Since the replication crisis, much effort has been made to improve our methodological practices, which is extremely encouraging. I hope that this aspiration toward more rigor will also be reflected in how we approach the theoretical foundations of our research.

## Author Contributions

The author confirms being the sole contributor of this work and has approved it for publication.

### Conflict of Interest Statement

The author declares that the research was conducted in the absence of any commercial or financial relationships that could be construed as a potential conflict of interest.
