# Supplementary material for: Evolutionary Theories and Men's Preferences for Women's Waist-to-Hip Ratio: Which Hypotheses Remain? A Systematic Review
Source: Front Psychol. 2019 Jun 4;10:1221. doi: 10.3389/fpsyg.2019.01221 (PMC6563790; doi:10.3389/fpsyg.2019.01221)
Supplement: Supplementary file 2 [file Data_Sheet_1.pdf]

frontiers

*Supplementary Material*

# Evolutionary theories and men's preferences for women's Waist-to-Hip Ratio: Which Hypotheses Remain? A systematic review.

**Jeanne Bovet***

*** Correspondence:** Jeanne Bovet: jeanne.bovet@gmail.com

## Collection methods

I searched for scientific articles and book chapters using Google Scholar. Searches included various combinations of the keywords: "WHR", "waist", "hip", "men's preferences", "human mate choice", "mating preferences" and "physical attractiveness". Next, an extensive review of the references of relevant articles was carried out. I only include published research where the primary language is English. Articles and book chapters are included if they address men's preferences for women's WHR, based on an evolutionary approach.

The final dataset consists of 104 papers from 58 different first authors, including 13 review papers and chapters, from 1993 to 2017. There are a few references to the adaptive role of preferences for curvaceousness or roundedness before 1993, but this collection starts with the first paper explicitly mentioning WHR as a cue of mate value (Singh, 1993a).

## Inclusion and exclusion criteria of the hypotheses

Hypotheses concerning men's adaptive preferences toward women's WHRs, waist size or hip size are included. Descriptive information about WHR (e.g., "WHR is sexually dimorphic", or "WHR decrease at puberty in women") and proximal mechanisms for a low or high WHR (e.g., effects of hormones on fat storage and distribution), are not listed as hypotheses for men's adaptive preferences (but they are included in figure 1 and supplementary table, for the record). Hypotheses concerning the evolutionary benefits of preferences for fat quantity (and not distribution) are excluded as they are not specific to WHR.

Hypotheses assuming that WHR is only an indicator of BMI are not included. According to this idea, preferences for a certain WHR would be an artefact of preferences for a certain BMI. This argument is mostly targeting specific studies using stimuli confounding the two measurements (for example, by increasing WHR through the manipulation of the waist only). It is true that WHR and BMI are correlated, but the correlation varies greatly according to the focus population (including or excluding pre-pubertal or post-menopausal women, pregnant, obese or anorexic women, etc. Sugiyama, 2015). Overall, there is enough evidence that WHR conveys information which is different from BMI (Sugiyama, 2015).

It is also important to make the distinction between hypotheses regarding the selection of men's preferences toward a certain WHR (what we are interested in here) and hypotheses about the evolutionary origin of a specific female WHR in the human species. The origin of human's female WHR and men's preference for female WHR can be related, but the link is not consequential (i.e., the

selection of a low WHR does not necessarily lead to a selection of the preferences for a low WHR). Hypotheses concerning the evolution of a specific WHR in humans by natural selection are not included, unless they are described as hypotheses for the selection of men's preferences as well.

Non-adaptive hypotheses are not included here (e.g., sociocultural explanations and the super-stimuli hypothesis; see the discussion section).

## Coding of the hypotheses

First, all the hypotheses used to explain an adaptive preference for a certain (low, high or average) WHR are listed. Then, each paper from the dataset is reviewed individually to see which of these hypotheses was mentioned (see supplementary table S1). The hypothesis is coded as present even if mentioned very briefly using a few words only. For example, the sentence "WHR is a cue of youth and fertility" is sufficient to validate the presence of the hypotheses "WHR as a cue of (reproductive) age" and "WHR as a cue of fertility or potential reproductive success" (see supplementary table S1). Synonyms are accounted for. For example, the terms "Reproductive capability" and "Reproductively viable" are recorded within the "WHR as a cue of fertility or potential reproductive success" hypothesis. To be coded as present, a hypothesis only needs to be mentioned, not endorsed by the author (the hypothesis is coded as present even when the author is conveying doubts about its plausibility, although this case is very rare).

In a few cases, references to a hypothesis are ambiguous. For example, the term "reproductive status" is used in two papers, without further explanation. This term is large and could, in theory, include disparate hypotheses such as "WHR as a cue of fertility or potential reproductive success", "WHR as a cue of current pregnancy", "WHR as a cue of (reproductive) age" and "WHR as a cue of parity", for example. Without any additional information nor references associated to the term, all the potential hypotheses are recorded as present (but indicated with an interrogation mark in supplementary table S1). Similarly, in one chapter (Little, 2015), "indirect benefits" are very briefly mentioned, which, at least in evolutionary biology, usually implies genetic quality (the hypothesis "cue of genetic quality" is recorded in that case).

**Supplementary Table S1. References to the different hypotheses of men's adaptive preferences for women's WHR in the literature in the past 25 years**. This table includes the papers collected for this review: scientific papers and chapters exploring the relationships between WHR and female attractiveness with an evolutionary perspective. In a few cases, the reference to the hypothesis was unclear and represented with an interrogation mark. Reviews are signaled with a striped background. Characteristics related to women's fertility are represented in blue. In green: characteristics related to women's health. In yellow: characteristics related to women's environment. In orange: characteristics related to the descendants' quality.

## References list for the dataset

Andrews, T. M., Lukaszewski, A. W., Simmons, Z. L., and Bleske-Rechek, A. (2017). Cue-based estimates of reproductive value explain women's body attractiveness. *Evol. Hum. Behav.* doi:10.1016/j.evolhumbehav.2017.04.002.

Bovet, J., Lao, J., Bartholomée, O., Caldara, R., and Raymond, M. (2016). Mapping female bodily features of attractiveness. *Scientific Reports* 6, 18551. doi:10.1038/srep18551.

Bovet, J., and Raymond, M. (2015). Preferred women's waist-to-hip ratio variation over the last 2,500 years. *PLOS ONE* 10, e0123284. doi:10.1371/journal.pone.0123284.

Brooks, R. C., Shelly, J. P., Jordan, L. A., and Dixson, B. J. (2015). The multivariate evolution of female body shape in an artificial digital ecosystem. *Evol. Hum. Behav.* 36, 351–358.

Brooks, R., Shelly, J. P., Fan, J., Zhai, L., and Chau, D. K. P. (2010). Much more than a ratio: multivariate selection on female bodies. *Journal of Evolutionary Biology* 23, 2238–2248. doi:10.1111/j.1420-9101.2010.02088.x.

Buss, D. M. (2003). *The Evolution Of Desire: Strategies of Human Mating*. Basic Books.

Buunk, B. P., and Dijkstra, P. (2005). A narrow waist versus broad shoulders: Sex and age differences in the jealousy-evoking characteristics of a rival's body build. *Pers. Individ. Differ.* 39, 379–389. doi:10.1016/j.paid.2005.01.020.

Cashdan, E. (2008). Waist-to-Hip Ratio across Cultures: Trade-Offs between Androgen- and Estrogen-Dependent Traits. *Current Anthropology* 49, 1099–1107. doi:10.1086/593036.

Cloud, J. M., and Perilloux, C. (2014). "Bodily Attractiveness as a Window to Women's Fertility and Reproductive Value," in *Evolutionary Perspectives on Human Sexual Psychology and Behavior* Evolutionary Psychology. (Springer, New York, NY), 135–152. doi:10.1007/978-1-4939-0314-6_7.

Cloud, J. M., and Perilloux, C. (2015). "Drawing" Conclusions About Perceptions of Ideal Male and Female Body Shapes. *Evolutionary Psychological Science* 1, 163–171.

Connolly, J. M., Slaughter, V., and Mealey, L. (2004). The development of preferences for specific body shapes. *J. Sex Res.* 41, 5–15.

Cornelissen, P. L., Hancock, P. J. B., Kiviniemi, V., George, H. R., and Tovée, M. J. (2009a). Patterns of eye movements when male and female observers judge female attractiveness, body fat and waist-to-hip ratio. *Evol. Hum. Behav.* 30, 417–428.

Cornelissen, P. L., Tovée, M. J., and Bateson, M. (2009b). Patterns of subcutaneous fat deposition and the relationship between body mass index and waist-to-hip ratio: Implications for models of physical attractiveness. *Journal of Theoretical Biology* 256, 343–350. doi:10.1016/j.jtbi.2008.09.041.

Crossley, K. L., Cornelissen, P. L., and Tovée, M. J. (2012). What Is an Attractive Body? Using an Interactive 3D Program to Create the Ideal Body for You and Your Partner. *PLoS One* 7. Available at: ://WOS:000312104900070.

Deady, D. K., and Smith, M. J. L. (2015). Changing Male Preferences for Female Body Type in the US: An Adaptive Response to a Changing Socioeconomic Climate. *Journal of Behavioral and Brain Science* 5, 570.

Dijkstra, P., and Buunk, B. P. (2001). Sex differences in the jealousy-evoking nature of a rival's body build. *Evol. Hum. Behav.* 22, 335–341. doi:10.1016/S1090-5138(01)00070-8.

Dixson, B. J., Dixson, A. F., Bishop, P. J., and Parish, A. (2010a). Human Physique and Sexual Attractiveness in Men and Women: A New Zealand–U.S. Comparative Study. *Arch. Sex. Behav.* 39, 798–806. doi:10.1007/s10508-008-9441-y.

Dixson, B. J., Dixson, A. F., Li, B. G., and Anderson, M. J. (2007a). Studies of human physique and sexual attractiveness: Sexual preferences of men and women in China. *Am. J. Hum. Biol.* 19, 88–95.

Dixson, B. J., Dixson, A. F., Morgan, B., and Anderson, M. J. (2007b). Human physique and sexual attractiveness: Sexual preferences of men and women in Bakossiland, Cameroon. *Arch. Sex. Behav.* 36, 369–375.

Dixson, B. J., Grimshaw, G. M., Linklater, W. L., and Dixson, A. F. (2009). Eye-Tracking of Men's Preferences for Waist-to-Hip Ratio and Breast Size of Women. *Arch. Sex. Behav.* 40, 43–50.

Dixson, B. J., Grimshaw, G. M., Linklater, W. L., and Dixson, A. F. (2010b). Watching the Hourglass Eye Tracking Reveals Men's Appreciation of the Female Form. *Human Nature-an Interdisciplinary Biosocial Perspective* 21, 355–370.

Dixson, B. J., Sagata, K., Linklater, W. L., and Dixson, A. F. (2010c). Male Preferences for Female Waist-to-hip Ratio and Body Mass Index in the Highlands of Papua New Guinea. *Am. J. Phys. Anthropol.* 141, 620–625.

Donohoe, M. L., von Hippel, W., and Brooks, R. C. (2009). Beyond waist-hip ratio: experimental multivariate evidence that average women's torsos are most attractive. *Behav. Ecol.* 20, 716–721.

Faries, M. D., and Bartholomew, J. B. (2012). The role of body fat in female attractiveness. *Evol. Hum. Behav.* 33, 672–681.

Fink, B., Klappauf, D., Brewer, G., and Shackelford, T. K. (2014). Female physical characteristics and intra-sexual competition in women. *Pers. Individ. Differ.* 58, 138–141. doi:10.1016/j.paid.2013.10.015.

Freedman, R. E. K., Carter, M. M., Sbrocco, T., and Gray, J. J. (2004). Ethnic differences in preferences for female weight and waist-to-hip ratio: A comparison of African–American and White American college and community samples. *Eating Behaviors* 5, 191–198. doi:10.1016/j.eatbeh.2004.01.002.

Furnham, A., Dias, M., and McClelland, A. (1998). The role of body weight, waist-to-hip ratio, and breast size in judgments of female attractiveness. *Sex Roles* 39, 311–326.

Furnham, A., Lavancy, M., and McClelland, A. (2001). Waist to hip ratio and facial attractiveness: a pilot study. *Personality and Individual Differences* 30, 491–502. doi:10.1016/S0191-8869(00)00040-4.

Furnham, A., McClelland, A., and Omer, L. (2003). A cross-cultural comparison of ratings of perceived fecundity and sexual attractiveness as a function of body weight and waist-to-hip ratio. *Psychology, Health & Medicine* 8, 219–2304.

Furnham, A., Mistry, D., and McClelland, A. (2004). The influence of age of the face and the waist to hip ratio on judgements of female attractiveness and traits. *Personality and Individual Differences* 36, 1171–1185. doi:10.1016/S0191-8869(03)00209-5.

Furnham, A., Moutafi, J., and Baguma, P. (2002). A cross-cultural study on the role of weight and waist-to-hip ratio on female attractiveness. *Personality and Individual Differences* 32, 729–745.

Furnham, A., and Nordling, R. (1998). Cross-cultural differences in preferences for specific male and female body shapes. *Personality and Individual Differences* 25, 635–648. doi:10.1016/S0191-8869(98)00076-2.

Furnham, A., Petrides, K. V., and Constantinides, A. (2005). The effects of body mass index and waist-to-hip ratio on ratings of female attractiveness, fecundity, and health. *Personality and Individual Differences* 38, 1823–1834. doi:10.1016/j.paid.2004.11.011.

Furnham, A., Swami, V., and Shah, K. (2006). Body weight, waist-to-hip ratio and breast size correlates of ratings of attractiveness and health. *Personality and Individual Differences* 41, 443–454.

Furnham, A., Tan, T., and McManus, C. (1997). Waist-to-hip ratio and preferences for body shape: A replication and extension. *Pers. Individ. Differ.* 22, 539–549.

Gallup Jr, G. G., and Frederick, D. A. (2010). The science of sex appeal: An evolutionary perspective. *Review of General Psychology* 14, 240.

Gangestad, S. W., and Scheyd, G. J. (2005). The evolution of human physical attractiveness. *Annu. Rev. Anthropol.* 34, 523–548. doi:10.1146/annurev.anthro.33.070203.143733.

Garza, R., Heredia, R. R., and Cieslicka, A. B. (2016). Male and Female Perception of Physical Attractiveness: An Eye Movement Study. *Evolutionary Psychology* 14. doi:10.1177/1474704916631614.

Grillot, R. L., Simmons, Z. L., Lukaszewski, A. W., and Roney, J. R. (2014). Hormonal and morphological predictors of women's body attractiveness. *Evol. Hum. Behav.* 35, 176–183. doi:10.1016/j.evolhumbehav.2014.01.001.

Henss, R. (1995). Waist-to-hip ratio and attractiveness. Replication and extension. *Personality and Individual Differences* 19, 479–488.

Henss, R. (2000). Waist-to-hip ratio and female attractiveness. Evidence from photographic stimuli and methodological considerations. *Personality and Individual Differences* 28, 501–513.

Holliday, I. E., Longe, O. A., Thai, N. J., Hancock, P. J. B., and Tovée, M. J. (2011). BMI Not WHR Modulates BOLD fMRI Responses in a Sub-Cortical Reward Network When Participants Judge the Attractiveness of Human Female Bodies. *PLOS ONE* 6. Available at: ://WOS:000297555300013.

Hudson, M. J., and Aoyama, M. (2007). Waist-to-hip ratios of Jomon figurines. *Antiquity* 81, 961–971.

Karremans, J. C., Frankenhuis, W. E., and Arons, S. (2010). Blind men prefer a low waist-to-hip ratio. *Evol. Hum. Behav.* 31, 182–186. doi:10.1016/j.evolhumbehav.2009.10.001.

Koscinski, K. (2013). Attractiveness of womens body: body mass index, waisthip ratio, and their relative importance. *Behav. Ecol.* 24, 914–925.

Kościński, K. (2014). Assessment of Waist-to-Hip Ratio Attractiveness in Women: An Anthropometric Analysis of Digital Silhouettes. *Arch. Sex. Behav.* 43, 989–997. doi:10.1007/s10508-013-0166-1.

Lassek, W. D., and Gaulin, S. J. C. (2016). What makes jessica rabbit sexy? Contrasting roles of waist and hip size. *Evolutionary Psychology* 14. doi:10.1177/1474704916643459.

Lee, A. J., Brooks, R. C., Potter, K. J., and Zietsch, B. P. (2015). Pathogen disgust sensitivity and resource scarcity are associated with mate preference for different waist-to-hip ratios, shoulder-to-hip ratios, and body mass index. *Evol. Hum. Behav.* 36, 480–488. doi:10.1016/j.evolhumbehav.2015.07.002.

Little, A. C. (2015). "Attraction and human mating," in *Evolutionary perspectives on social psychology* (Springer), 319–332.

Manning, J. T., Trivers, R. L., Singh, D., and Thornhill, R. (1999). The mystery of female beauty. *Nature* 399, 214–215. doi:10.1038/20341.

Marlowe, F. W., Apicella, C., and Reed, D. (2005). Men's preferences for women's profile waist-to-hip ratio in two societies. *Evol. Hum. Behav.* 26, 458–468.

Marlowe, F. W., and Wetsman, A. (2001). Preferred waist-to-hip ratio and ecology. *Pers. Individ. Differ.* 30, 481–489.

Miller, G. F., and Todd, P. M. (1998). Mate choice turns cognitive. *Trends Cogn. Sci.* 2, 190–198.

Pawłowski, B., and Dunbar, R. I. M. (2005). Waist-to-hip ratio versus body mass index as predictors of fitness in women. *Hum Nat* 16, 164–177. doi:10.1007/s12110-005-1002-7.

Pettijohn, T. F., and Jungeberg, B. J. (2004). Playboy Playmate curves: Changes in facial and body feature preferences across social and economic conditions. *Pers. Soc. Psychol. Bull.* 30, 1186–1197.

Platek, S. M., and Singh, D. (2010). Optimal Waist-to-Hip Ratios in Women Activate Neural Reward Centers in Men. *PLoS One* 5. Available at: ://WOS:000274442400002.

Pokrywka, L., Čabrić, M., and Krakowiak, H. (2006). Body Mass Index and Waist: Hip Ratio are Not Enough to Characterise Female Attractiveness. *Perception* 35, 1693–1697. doi:10.1068/p5506.

Price, M. E., Pound, N., Dunn, J., Hopkins, S., and Kang, J. S. (2013). Body Shape Preferences: Associations with Rater Body Shape and Sociosexuality. *PLOS ONE* 8.

Rilling, J. K., Kaufman, T. L., Smith, E. O., Patel, R., and Worthman, C. M. (2009). Abdominal depth and waist circumference as influential determinants of human female attractiveness. *Evol. Hum. Behav.* 30, 21–31.

Rozmus-Wrzesinska, M., and Pawlowski, B. (2005). Men's ratings of female attractiveness are influenced more by changes in female waist size compared with changes in hip size. *Biol. Psychol.* 68, 299–308.

Schaefer, K., Fink, B., Grammer, K., Mitteroecker, P., Gunz, P., and Bookstein, F. L. (2006). Female appearance: facial and bodily attractiveness as shape. *Psychology Science* 48, 187–204.

Schützwohl, A. (2006). Judging female figures: A new methodological approach to male attractiveness judgments of female waist-to-hip ratio. *Biol. Psychol.* 71, 223–229. doi:10.1016/j.biopsycho.2005.04.005.

Shackleford, T., and Weekes-Shackleford, V. (2017). *Encyclopedia of Evolutionary Psychological Science*. Springer.

Singh, D. (1993a). Adaptive significance of female physical attractiveness - Role of waist-to-hip ratio. *J. Pers. Soc. Psychol.* 65, 293–307.

Singh, D. (1993b). Body shape and women's attractiveness - The critical role of waist-to-hip ratio. *Hum. Nat.-Interdiscip. Biosoc. Perspect.* 4, 297–321.

Singh, D. (1994). Is thin really beautiful and good - Relationship between waist-to-hip ratio (WHR) and female attractiveness. *Pers. Individ. Differ.* 16, 123–132.

Singh, D. (1995). Female health, attractiveness, and desirability for relationships: Role of breast asymmetry and waist-to-hip ratio. *Ethology and Sociobiology* 16, 465–481.

Singh, D., Dixson, B. J., Jessop, T. S., Morgan, B., and Dixson, A. F. (2010). Cross-cultural consensus for waist-hip ratio and women's attractiveness. *Evol. Hum. Behav.* 31, 176–181.

Singh, D., and Luis, S. (1995). Ethnic and gender consensus for the effect of waist-to-hip ratio on judgment of women's attractiveness. *Hum. Nat.-Interdiscip. Biosoc. Perspect.* 6, 51–65.

Singh, D., and Randall, P. K. (2007). Beauty is in the eye of the plastic surgeon: Waist-hip ratio (WHR) and women's attractiveness. *Pers. Individ. Differ.* 43, 329–340. doi:10.1016/j.paid.2006.12.003.

Singh, D., and Singh, D. (2011). Shape and Significance of Feminine Beauty: An Evolutionary Perspective. *Sex Roles* 64, 723–731.

Singh, D., and Young, R. K. (1995). Body weight, waist-to-hip ratio, breasts, and hips: Role in judgments of female attractiveness and desirability for relationships. *Ethology and Sociobiology* 16, 483–507.

Smith, K. L., Cornelissen, P. L., and Tovée, M. J. (2007). Color 3D bodies and judgements of human female attractiveness. *Evol. Hum. Behav.* 28, 48–54. doi:10.1016/j.evolhumbehav.2006.05.007.

Sorokowski, P., Kościński, K., Sorokowska, A., and Huanca, T. (2014). Preference for women's body mass and waist-to-hip ratio in tsimane'men of the bolivian amazon: biological and cultural determinants. *PLOS ONE* 9, e105468.

Sorokowski, P., and Sorokowska, A. (2012). Judgments of Sexual Attractiveness: A Study of the Yali Tribe in Papua. *Arch. Sex. Behav.* 41, 1209–1218.

Streeter, S. A., and McBurney, D. H. (2003). Waist-hip ratio and attractiveness - New evidence and a critique of "a critical test." *Evol. Hum. Behav.* 24, 88–98.

Sugiyama, L. S. (2004). Is beauty in the context-sensitive adaptations of the beholder? Shiwiar use of waist-to-hip ratio in assessments of female mate value. *Evol. Hum. Behav.* 25, 51–62.

Sugiyama, L. S. (2005). "Physical attractiveness: an adaptationist perspective," in *The Handbook of Evolutionary Psychology* (Hoboken, New Jersey: John Wiley & Sons, Inc.), 292–343.

Sugiyama, L. S. (2015). "Physical attractiveness: an adaptationist perspective," in *The Handbook of Evolutionary Psychology* (Hoboken, New Jersey: John Wiley & Sons, Inc.), 317–384.

Suschinsky, K. D., Elias, L. J., and Krupp, D. B. (2007). Looking for Ms. Right: Allocating attention to facilitate mate choice decisions. *Evolutionary Psychology* 5, 428–441.

Swami, V., Antonakopoulos, N., Tovée, M. J., and Furnham, A. (2006a). A critical test of the waist-to-hip ratio hypothesis of women's physical attractiveness in Britain and Greece. *Sex Roles* 54, 201–211.

Swami, V., Caprario, C., Tovée, M. J., and Furnham, A. (2006b). Female physical attractiveness in Britain and Japan: A cross-cultural study. *Eur. J. Personal.* 20, 69–81.

Swami, V., Grant, N., Furnham, A., and McManus, I. C. (2007a). Perfectly formed? The effect of manipulating the waist-to-hip ratios of famous paintings and sculptures. *Imagination, Cognition and Personality* 27, 47–62.

Swami, V., Gray, M., and Furnham, A. (2007b). The female nude in Rubens: disconfirmatory evidence of the waist-to-hip ratio hypothesis of female physical attractivenes. *Imagination, Cognition and Personality* 26, 139–147.

Swami, V., Jones, J., Einon, D., and Furnham, A. (2009). Men's preferences for women's profile waist-to-hip ratio, breast size, and ethnic group in Britain and South Africa. *Br. J. Psychol.* 100, 313–325.

Swami, V., Neto, F., Tovée, M. J., and Furnham, A. (2007c). Preferences for Female Body Weight and Shape in Three European Countries. *European Psychologist* 12, 220–228. doi:10.1027/1016-9040.12.3.220.

Swami, V., and Tovée, M. (2007a). Differences in attractiveness preferences between observers in low- and high-resource environments in Thailand. *Journal of Evolutionary Psychology* 5, 149–160.

Swami, V., and Tovée, M. J. (2005). Female physical attractiveness in Britain and Malaysia: A cross-cultural study. *Body Image* 2, 115–128. doi:10.1016/j.bodyim.2005.02.002.

Swami, V., and Tovée, M. J. (2006). Does hunger influence judgments of female physical attractiveness? *British Journal of Psychology* 97, 353–363. doi:10.1348/000712605X80713.

Swami, V., and Tovée, M. J. (2007b). Perceptions of female body weight and shape among indigenous and urban Europeans. *Scandinavian Journal of Psychology* 48, 43–50. doi:10.1111/j.1467-9450.2006.00526.x.

Swami, V., and Tovée, M. J. (2007c). The relative contribution of profile body shape and weight to judgements of women's physical attractiveness in Britain and Malaysia. *Body Image* 4, 391–396.

Tassinary, L. G., and Hansen, K. A. (1998). A critical test of the waist-to-hip-ratio hypothesis of female physical attractiveness. *Psychol. Sci.* 9, 150–155.

Tovée, M. J., and Cohen-Tovée, E. M. (2016). "Body Fat Percent and Distribution," in *Encyclopedia of Evolutionary Psychological Science*, eds. V. Weekes-Shackelford, T. K. Shackelford, and V. A. Weekes-Shackelford (Springer International Publishing), 1–6. doi:10.1007/978-3-319-16999-6_86-1.

Tovée, M. J., Furnham, A., and Swami, V. (2007). "Healthy body equals beautiful body? Changing perceptions of health and attractiveness with shifting socioeconomic status," in *The Body Beautiful*, eds. V. Swami and A. Furnham (Palgrave Macmillan UK), 108–128. doi:10.1057/9780230596887_7.

Tovée, M. J., Hancock, P. J. B., Mahmoodi, S., Singleton, B. R. R., and Cornelissen, P. L. (2002). Human female attractiveness: waveform analysis of body shape. *Proc. R. Soc. B* 269, 2205–2213. doi:10.1098/rspb.2002.2133.

Tovée, M. J., Maisey, D. S., Emery, J. L., and Cornelissen, P. L. (1999). Visual cues to female physical attractiveness. *Proc. R. Soc. B* 266, 211–218.

Tovée, M. J., Swami, V., Furnham, A., and Mangalparsad, R. (2006). Changing perceptions of attractiveness as observers are exposed to a different culture. *Evol. Hum. Behav.* 27, 443–456.

Voland, E., and Grammer, K. (2013). *Evolutionary Aesthetics*. Springer Science & Business Media.

Voracek, M., and Fisher, M. L. (2002). Shapely centrefolds? Temporal change in body measures: trend analysis. *BMJ: British Medical Journal* 325, 1447.

Voracek, M., and Fisher, M. L. (2006). Success is all in the measures: Androgenousness, curvaceousness, and starring frequencies in adult media actresses. *Arch. Sex. Behav.* 35, 297–304.

Wang, G., Djafarian, K., Egedigwe, C. A., El Hamdouchi, A., Ojiambo, R., Ramuth, H., et al. (2015). The relationship of female physical attractiveness to body fatness. *PeerJ* 3, e1155.

Wetsman, A., and Marlowe, F. W. (1999). How universal are preferences for female waist-to-hip ratios? Evidence from the Hadza of Tanzania. *Evol. Hum. Behav.* 20, 219–228.

Yu, D. W., and Shepard Jr., G. H. (1998). Is beauty in the eye of the beholder? *Nature* 396, 321–322.

Zotto, M. D., and Pegna, A. J. (2017). Electrophysiological evidence of perceived sexual attractiveness for human female bodies varying in waist-to-hip ratio. *Cogn Affect Behav Neurosci* 17, 577–591. doi:10.3758/s13415-017-0498-8.
